# Mobile technology supporting trainee doctors’ workplace learning and patient care: an evaluation

**DOI:** 10.1186/1472-6920-13-6

**Published:** 2013-01-21

**Authors:** Wendy Hardyman, Alison Bullock, Alice Brown, Sophie Carter-Ingram, Mark Stacey

**Affiliations:** 1 Cardiff Unit for Research and Evaluation in Medical and Dental Education, Cardiff University School of Social Sciences, 2nd Floor, Glamorgan Building, King Edward VII Avenue, Cathays Park, Cardiff CF10 3WT, UK; 2 ACCS, London Deanery, London, UK; 3 Wales Deanery, School of Postgraduate Medical and Dental Education, Cardiff University, 9th Floor, Neuadd Meirionnydd, Heath Park, Cardiff, CF14 4YS, UK

**Keywords:** Technology enhanced learning, Workplace learning, Workplace information source, Trainee doctors, Smartphones, Transitions

## Abstract

**Background:**

The amount of information needed by doctors has exploded. The nature of knowledge (explicit and tacit) and processes of knowledge acquisition and participation are complex. Aiming to assist workplace learning, Wales Deanery funded “iDoc”, a project offering trainee doctors a Smartphone library of medical textbooks.

**Methods:**

Data on trainee doctors’ (Foundation Year 2) workplace information seeking practice was collected by questionnaire in 2011 (n = 260). iDoc baseline questionnaires (n = 193) collected data on Smartphone usage alongside other workplace information sources. Case reports (n = 117) detail specific instances of Smartphone use.

**Results:**

Most frequently (daily) used information sources in the workplace: senior medical staff (80% F2 survey; 79% iDoc baseline); peers (70%; 58%); and other medical/nursing team staff (53% both datasets). Smartphones were used more frequently by males (p < 0.01). Foundation Year 1 (newly qualified) was judged the most useful time to have a Smartphone library because of increased responsibility and lack of knowledge/experience.

Preferred information source varied by question type: hard copy texts for information-based questions; varied resources for skills queries; and seniors for more complex problems. Case reports showed mobile technology used for simple (information-based), complex (problem-based) clinical questions and clinical procedures (skills-based scenarios). From thematic analysis, the Smartphone library assisted: teaching and learning from observation; transition from medical student to new doctor; trainee doctors’ discussions with seniors; independent practice; patient care; and this ‘just-in-time’ access to reliable information supported confident and efficient decision-making.

**Conclusion:**

A variety of information sources are used regularly in the workplace. Colleagues are used daily but seniors are not always available. During transitions, constant access to the electronic library was valued. It helped prepare trainee doctors for discussions with their seniors, assisting the interchange between explicit and tacit knowledge.

By supporting accurate prescribing and treatment planning, the electronic library contributed to enhanced patient care. Trainees were more rapidly able to medicate patients to reduce pain and more quickly call for specific assessments. However, clinical decision-making often requires dialogue: what Smartphone technology can do is augment, not replace, discussion with their colleagues in the community of practice.

## Background

In adopting an evidence-based approach to patient treatment, doctors need to negotiate an abundance of information. The amount of medical information needed by practitioners has exploded and the complexity of treatment for many patients requires a tailored approach [1]. Davies [2] suggested that information from biomedical research doubles every twenty years and argued that it is “virtually impossible for medical staff to retain all the knowledge required to treat all the patients they examine over the course of their careers”. This knowledge explosion, coupled with a reduction in patient contact time as a result of the European Working Time Directive (EWTD), can leave trainees in need of additional support, particularly at times of transition which are known to be associated with heightened risk of unwelcome outcomes [3]. Transition from medical school to clinical practice has been associated with increased patient mortality [4] and described as “notoriously difficult” [5].

Work by Weightman and Williamson [6] demonstrated improvements in patient care, diagnosis, choice of therapy and tests and also reduction in hospital stay when professional library services were used. It is now possible for Smartphones to provide immediate electronic access to medical texts without the need of the internet. Software can allow users to search quickly across a library of texts. Such technology offers point-of-care, bedside or ‘just-in-time’ information to support learning and practice. Despite many studies alluding to these potential benefits, the use of mobile technology providing immediate access to electronic resources has undergone limited evaluation in workplace settings in healthcare [7] although a small number of recent studies have explored the educational use of personal digital assistants (PDAs) preloaded with medical textbooks [8,9].

This paper reports an evaluation of the “iDoc” initiative which provided trainee doctors in Wales with a library of texts on a Smartphone. Within a wider context of use of information sources in the workplace, the evaluation sought to find out how, when and why the Smartphone library was used and the outcomes and impact on knowledge and practice. We begin by setting out the background theoretical context, exploring the nature of knowledge and knowledge conversion and connections between knowledge and activity in a social community of practice [10]. The main body of the paper reports and discusses findings from the iDoc evaluation.

### Information and knowledge

Distinction has been drawn between two main forms of knowledge – explicit and tacit. Explicit knowledge is codified and can be expressed in formal, declarative language (for example, as in text books). Tacit knowledge, essential to the professional practitioner, is hard to communicate in words but developed through action and experience. Nonaka [11] described the interactions between the different forms of knowledge, identifying four distinct processes of “knowledge conversion”. Firstly, Nonaka [11] argued that the conversion of tacit knowledge to new tacit knowledge occurs through a process of *socialization* (sharing experiences through spending time together, in and outside the workplace and through apprenticeship). Secondly, the conversion of tacit knowledge to explicit knowledge is described as a process of *externalization* whereby the tacit knowledge is articulated in a way that enables it to be shared by others. This is hard to do but is assisted by the use of stories and metaphors and by repeated and extended dialogue and discussion. Thirdly, *combination* is the process of creating new explicit knowledge by, for example, classifying or reconfiguring existing explicit knowledge. The fourth process described is that of *internalization* through which explicit knowledge is shared and converted (internalized) into tacit knowledge. According to Nonaka [11] internalization occurs through action and experimentation.

A library of texts on a Smartphone provides access to explicit knowledge. There is potential that such ready access to information could support the process of internalization and the development of tacit knowledge [11]. As explicit and tacit knowledge are complementary, development of one helps in the development of the other. This is important in medical education because, it is argued, so much of doctors’ knowledge is implicit or tacit [12].

### Learning processes

The nature of knowledge and knowledge conversion processes can be directly related to ideas about learning. Two terms in use in the discourse about learning are ‘acquisition’ and ‘participation’ [13]. In an acquisition model, emphasis is given to the individual mind and what goes into it. In contrast, the participation model focuses on the bonds between individuals and others and learning is conceived more as a process of becoming a member of a particular community. In this model learning is ongoing and not separable from context. Table 1 sets out contrasts between these two models of learning.

**Table 1:** Contrasts between acquisition and participation models of learning (after Sfard, 1998)

	**Acquisition**	**Participation**
Goal of learning	Individual enrichment	Community building
Learning	Acquisition of something	Becoming a participant
Student	Recipient (consumer), re-constructor	Peripheral participant, apprentice
Teacher	Provider, facilitator, mediator	Expert participant, preserver of practice/discourse
Knowledge, concept	Property, possession, commodity	Aspects of practice/discourse/activity
Knowing	Having, possessing	Belonging, participating, communicating

Sfard [13] argued that acquisition and participation are not mutually exclusive and that it is neither desirable nor possible to ignore the place of acquisition in learning development; learners need to both acquire knowledge and participate in learning processes. In relation to participation, we note here the notion of ‘communities of practice’ from the work of Lave and Wenger [10]. Their ideas are pertinent to trainee doctors’ learning in the workplace and the role of communities of practice in learning development. Lave and Wenger [10] argue that to become a member of the community involves much more than the technical knowledge or skills associated with undertaking certain tasks. Members of the community are involved in a set of relationships over time which gives rise to a shared sense of enterprise and identity. Thus learning is seen as not only about the acquisition of knowledge but also as a process of social participation.

In this introductory section we have sought to argue that knowledge is not stable; it is in constant flux and the quantity of knowledge seems to be increasing rapidly. The nature of knowledge is complex, and explicit and tacit forms of knowledge can be converted through processes of externalization, internalization, socialization and combination [11]. Knowledge acquisition and participation are both essential elements of learning processes [13] and there is an intimate connection between knowledge and activity in a social community of practice [10].

## Method

### The iDoc initiative

The iDoc project was an initiative funded by the Wales Deanery. It aimed to augment workplace learning by providing trainee doctors with rapid access to reliable information relevant for practice, using mobile phone technology. Targeted at Foundation trainees, the initiative also sought to support the transition from medical school to clinical practice. Foundation training in the UK is the two-year period of workplace training immediately following graduation from medical school.

In 2009/10, trainee doctors in Wales were offered a Smartphone device (HTC TYTNII) and a micro secure digital (SD) card pre-loaded with a software application (provided by Medhand International AB) containing a library of 17 medical textbooks including the *British National Formulary* (BNF), the *Oxford Handbook of Clinical Medicine,* the *Oxford Handbook of the Foundation Programme* and Netter’s *Atlas of Human Anatomy*. All texts were included in Medhand’s Universal Mobile Library (known as *Dr Companion*). Integrated into the library of texts is an electronic application (*DocTool Cross Library Search Tool*) which enables rapid searching across all books [8]. Full details of textbooks are provided in the Additional file 1.

Costs are related to four main factors: the duration of the licence (the length of time that the books are available); the number of textbooks included; the number of participants; and whether a device is provided. The study reported in this paper was costly as trainees were provided with mobile devices. To provide an indication of current costs, in 2012, 440 trainees across Wales were provided with a 12-month licence key, giving access to six medical textbooks, for use on their own android or iPhone at a cost of approximately £120 per trainee.

### Recruitment to iDoc

Recruitment to the iDoc project was optional. In the pilot phase (October 2009 to March 2010), invitation by e-mail was sent to the August 2009 intake of Foundation Year 1 (F1) trainees. In the main phase, (recruited mainly between December 2010 and April 2011), the invitation to participate was extended to all foundation trainee doctors in the Wales Deanery in both Foundation years 1 and 2 (F1 and F2).

Participants recruited to the main phase of the project were provided with ‘unlocked’ phones, which could be used with any network provider. This was a change to the pilot phase (where trainees had also been offered a prepaid call/internet/text message package) and in response to feedback from the pilot which suggested that barriers to recruitment included trainees being tied into existing phone contracts and not wishing to carry two phones, insurance costs, lack of face-to-face contact with the iDoc project team and the challenge of setting up a new phone and software when also starting a new job.

### iDoc evaluation

Building on an earlier published study which used PDA devices [8], the iDoc evaluation aimed to assess the value and impact of the Smartphone library of texts to support workplace learning. Research ethics approval was obtained from Cardiff University (PGMDE 02/12/2010). This paper reports on the evaluation of the main phase of the iDoc project (excluding the pilot).

In design, the evaluation drew on Ellaway’s [14] framework of factors to explore contextual mobile learning and David et al’s [15] approach (what works, how, when?). The evaluation collected narrative accounts (case reports) of the use of technology in action [16], within the broader context of use of information sources in the workplace. The evaluation had two strands.

*Strand 1 (iDoc questionnaires* and *case reports).* At the point of recruitment participants completed a baseline questionnaire which collected data on frequency, type, usefulness and variation in use of workplace information sources, including mobile devices providing access to electronic resources. Exit questionnaires were completed by participants from both phases. The design of these questionnaires was informed by the focus group discussions noted above and included a mix of open and closed questions. Questionnaires were confidential but not anonymous.

Participants also submitted narrative case reports detailing specific instances of usage of the Smartphone library of texts in action. Case reports were prepared using a pro forma designed for the purpose, with the headings: title, setting/context, problem or issue addressed, what happened, and reflections.

*Strand 2 (F2 information-seeking practice survey;* referred to as the *F2 survey).* To explore trainees’ workplace information-seeking practice, questionnaires were distributed to 260 F2s attending compulsory study days in six sites across Wales in early 2011. Most questions were closed.

### Analysis

Data from all questionnaires were entered into SPSS v18. All variable frequencies were reviewed. To explore relationships between variables, cross tabulations were performed and the chi squared test of significance applied. Responses to open questions were thematically analysed and main themes were agreed by two members of the research team. These themes were then assigned numerical codes and added to the SPSS database.

Case reports were analysed using NVivo, a qualitative data analysis software package. Data were first categorised by scenario-type. This classification distinguished three types of scenario: information-seeking, skills-based, and problem-based. These scenario-types were also utilised in the F2 survey. The classification of case reports by scenario-type was checked by a clinical member of the project team (consultant anaesthetist). Further analysis revealed additional sub-themes.

## Results

Data reported here are from baseline questionnaires (n = 193), the 2011 F2 survey (n = 260) and case reports collected in the period May 2011 to July 2012 (n = 117).

### Recruitment and questionnaire returns

In the main phase, 193 participants were recruited from 18 hospital sites mainly from December 2010 to April 2011. A few joined later (up to September 2011, n = 22). In the main phase participants comprised: F1 trainees (n = 95, 49%), F2s (n = 40, 21%) and a mixture of other training levels (30%, n = 58) including medical students (n = 20, 17 fifth years, 3 fourth years), clinical fellows (n = 2), core trainees, grades 1–3 (n = 25), specialist trainees, grades (n = 9) and specialist registrars (n = 2).

Two hundred and sixty trainees out of a possible 346 training grade places completed the anonymous information seeking practice questionnaire representing a 75% response rate (58% female, n = 150).

### Lack of knowledge during a patient consultation

Both the iDoc baseline questionnaire and the F2 survey asked respondents to select from four responses to indicate how they would respond if they were in a consultation with a patient and did not know something. The results are reported in Table 2. These indicate that in both groups the majority of respondents would inform the patient and seek information later.

**Table 2:** Information seeking behaviour when in a patient consultation

***When in consultation with a patient if you did not know something would you most likely*….’**	***iDoc baseline %***	***F2 survey %***
	***(number)***	***(number)***
1. Inform patient and seek information in front of them	27.5% (53)	21.7% (56)
2. Inform patient and seek information later	65.3% (126)	69.8% (180)
3. Inform patient and do nothing	0.0% (0)	0.8 % (2)
4. Not inform patient and seek information later	4.1% (8)	6.6% (17)
Selected option 1 and 2	3.1% (6)	1.2% (3)
TOTAL	100% (193)	100% (258)

### Use of workplace information sources

From the F2 survey and iDoc baseline data, the most frequently used information sources in the workplace *on a daily basis* were: senior medical staff (79% iDoc baseline dataset; 80% F2 dataset); peers (58%; 70%); and other staff in the medical/nursing team (53% both datasets). Electronic textbooks/journals were accessed using a *mobile device* at least weekly by 32%/34% (iDoc baseline/F2 survey). Mobile devices were used more frequently by males. This difference was statistically significant in both datasets (p < 0.01 chi.sq). Table 3 shows the frequency of workplace use of different information sources. Figures noted above are highlighted in bold.

**Table 3:** Use of information sources in the workplace

***Number***	***Information source***	***Never***	***Rarely***	***Monthly***	***Weekly***	***Daily***
		***% (n)***	***% (n)***	***% (n)***	***% (n)***	***% (n)***
*iDoc baseline *(n = 190)	Hardcopy texts/Journals	1.6 (3)	12.6 (24)	13.6 (26)	37.7 (72)	34 (65)
*F2 survey *(n = 251)		6.8 (17)	16.3 (41)	19.1 (48)	37.5 (94)	20.3 (51)
*iDoc baseline *(n = 185)	Electext/journal mobile device	38.4 (71)	19.5 (36)	9.7 (18)	**18.9 (35)**	**13.5 (25)**
*F2 survey *(n = 247)		31.2 (77)	24.3 (60)	10.9 (27)	**22.3 (55)**	**11.3 (28)**
*iDoc baseline *(n = 184)	Electext/journal PC	11.4 (21)	22.3 (41)	23.4 (43)	28.8 (53)	14.1 (26)
*F2 survey *(n = 249)		12.9 (32)	20.5 (51)	27.3 (68)	26.9 (67)	12.4 (31)
*iDoc baseline *(n = 183)	Lecture notes	44.3 (81)	34.4 (63)	12.6 (23)	7.1 (13)	1.6 (3)
*F2 survey *(n = 241)		56.8 (137)	33.2 (80)	7.1 (17)	2.9 (7)	0
*iDoc baseline *(n = 192)	Internet	0	3.1 (6)	8.9 (17)	43.8 (84)	44.3 (85)
*F2 survey *(n = 254)		1.2 (3)	3.5 (9)	5.9 (15)	47.6 (121)	41.7 (106)
*iDoc baseline *(n = 187)	Peers	0.5 (1)	2.1 (4)	2.1 (4)	37.4 (70)	**57.8 (108)**
*F2 survey *(n = 249)		0.8 (2)	1.6 (4)	4.4 (11)	23.7 (59)	**69.5 (173)**
*iDoc baseline *(n = 193)	Seniors	0	1.1 (1)	0.5 (37)	19.5 (150)	**78.9 (190)**
*F2 survey *(n = 255)		0.4 (1)	1.6 (4)	1.6 (4)	16.9 (43)	**79.6 (203)**
*iDoc baseline *(n = 183)	Other staff nursing/medical team	0.5 (1)	2.2% (4)	8.2 (15)	35.5 (67)	**52.5 (96)**
*F2 survey *(n = 248)		0.4 (1)	4.8 (12)	8.5 (21)	33.5 (83)	**52.8 (131)**

Electext = electronic text.

A small number of participants (n = 8 from iDoc baseline; n = 15 from F2 survey) included ‘other’ information sources, such as Trust Guidelines on intranet.

### Preferred information sources

iDoc participants at baseline were asked which of the listed resources was their preferred information source in the workplace. Seniors were reported as the most popular information resource (45%) followed by the internet (29%) and hardcopy textbooks/journals (16% plus 10% not specifying whether hard copy or electronic textbooks/journals). Respondents offered reasons for their preference of information source in the workplace. Main reasons related to ease and speed of access and the perceived reliability of the information source. Comments in relation to senior medical staff also focused on experience and application of information in context.

The F2 survey explored preferred information source across 12 specialities and by supervision level (directly or remotely supervised). Results indicated that in nine specialties, senior medical staff were the preferred information source when *directly* supervised. Hardcopy textbooks were the preferred information source when *remotely* supervised (9/12 specialities). The Internet was the *third* preferred information source across all specialities and at both levels of supervision.

### Types of information accessed in the workplace

On a daily basis the types of information the majority of respondents to the iDoc baseline needed to access in the workplace were drug dosage (n = 145; 76%) followed by treatment regimens (n = 100; 52%) and diagnosis (n = 69; 36%). Small numbers of respondents identified that they needed to access research evidence, anatomy and physiology related information on a daily basis (n = 11,6%; n = 15,8%; n = 18, 9% respectively), compared to accessing this information on a weekly basis (n = 78, 41%; n = 68, 36%; n = 71, 37% respectively).

### Variation in resource choice according to problem type

Findings from the F2 survey indicate that preference for information source depended on the nature of the issue/question. Three hypothetical scenarios were presented and preference varied by problem-type. The information-based scenario (which asked about finding the correct dose of chloperazine) showed preference for hardcopy textbooks/journals (89%); the problem-based scenario (about investigating a patient who had lost 5 stone in 5 months) showed preference for senior medical staff (87%); the skill-based scenario (asking about the structures a needle passes through when performing lumbar puncture) showed preference for hardcopy texts, seniors and internet (75%; 74%; 70%). Table 4 reports first, second and third choice of information source for each scenario based on total number of responses.

**Table 4:** Choice of information source by hypothetical scenarios (F2 survey)

***Information Source***	**Scenario 1**	**Scenario 2**	**Scenario 3**
	***Information-based % (n)***	***Skills-based % (n)***	***Problem-based % (n)***
Hard copy texts/journals	89% (230)	75% (194)	63% (164)
Seniors	67% (174)	74% (191)	87% (226)
Internet	56% (145)	70% (183)	65% (169)

### Views on when a mobile device containing texts would be most useful

Both the iDoc baseline and the F2 survey included questions about the stage in their medical career when a mobile device containing reliable information would be *most* useful. F1 (newly qualified) was judged to be the most useful time to have such devices (54% iDoc baseline; 43% F2 survey). These data are reported in full in Table 5. We note that although respondents were advised to tick one box, some respondents ticked more than one. Most common ‘other’ responses related to ‘training being lifelong’ and ‘all grades’ of training.

**Table 5:** Training grade when a mobile device was thought to be most useful

***Training grade***	***iDoc baseline***	***F2 survey***
	***(n = 192)% (n)***	***(n = 229)% (n)***
1. Undergraduate	13.0% (25)	23.1% (53)
2. F1	54.2% (104)	43.2% (99)
3. F2	5.7% (11)	7.9% (18)
4. Other	7.3% (14)	4.4% (10)
Mixed combinations	19.8% (38)	21.4% (49)

Respondents were asked to provide the main reasons for their selection of training grade. Responses were provided by 86% (iDoc baseline) and 67% (F2 survey) of participants. Across both datasets the main reasons given related to: increased responsibility, lack of knowledge, lack of experience, needing quick answers, needing most help, still learning, having time to read and early adoption of the technology. These responses were explored in relation to the level of training grade selected and are reported in Table 6.

**Table 6:** Main reasons why a mobile device would be useful at selected stage of training

***Grade selected***	**Increased responsibility**		**Lack of knowledge**		**Lack of experience**		**Need quick answers**		**Need most help**		**Still learning**		**Time to read**		**Early adoption**	
***Dataset***	**iDoc**	**F2**	**iDoc**	**F2**	**iDoc**	**F2**	**iDoc**	**F2**	**iDoc**	**F2**	**iDoc**	**F2**	**iDoc**	**F2**	**iDoc**	**F2**
*Under graduate*	0	0	6	3	2	0	2	3	2	0	1	0	7	14	1	9
*F1*	26	22	22	10	21	10	21	10	15	11	3	3	0	0	2	1
*F2*	6	9	1	0	0	0	2	5	0	0	1	0	0	0	0	0
*Mixed*	2	4	9	5	2	2	9	5	2	0	18	10	0	2	0	1
**Total**	**34**	**35**	**38**	**18**	**25**	**12**	**34**	**23**	**17**	**11**	**23**	**13**	**7**	**16**	**3**	**11**

The responses show that most reasons given for identifying the F1 as a time when a library of texts on a Smartphone would be most useful related to increased responsibility, lack of knowledge and experience, and a time of needing quick answers and most help. Example comments include:

It [F1] is the first point at which we are expected to know answers, often on our own, with senior support not always available…

Very important that F1s have access to resources to confirm and double check their knowledge as they are only newly qualified and still learning a great deal on the job.

### iDoc case reports

Case reports on experiences of using iDoc were received from 57 participants between May 2011 and July 2012. Most submitted more than one, providing a total of 117 reports. These reports were submitted mainly by foundation doctors (n = 47). The others ranged from 4th year medical students to specialist training year 3 (ST3) grade doctors.

The case reports were first classified by scenario-type. The majority related to either an information-based problem (n = 48) or were classified as problem-based (n = 52); seven were skills-based; and the remainder addressed wider questions. To illustrate, one information-based problem related to safe prescribing and dose adjustments in renal failure. In this example, the F1 trainee reflected:

The iDoc enabled me to quickly prescribe the necessary analgesia safely. The A&E department is extremely busy, so to wait for advice from a senior would have kept the patient waiting in pain for longer than was necessary with the use of the iDoc.

In a skills-based example, an F1 trainee reported the use of the iDoc device (Smartphone library) in obtaining guidance for setting up a urinary catheter. This trainee found “a step-by-step guide of the preparation and considerations for the procedure” which enabled him to “quickly and easily refresh my knowledge and prepare for the procedure thereby saving valuable time and improving patient care”.

One problem-based example described using the Smartphone library to assist in the diagnosis of the cause of an extensive blistering rash. This F1 trainee reported that:

With the rest of the senior members busy, the iDoc was invaluable in giving me a working diagnosis of Stevens–Johnson syndrome and suggesting a management plan and possible complications that I should be aware of…. [N]ot only did it help me initiate management to stabilise the patient but also directed me in organising further care by requesting a dermatology review.

Beyond the classification of case report by scenario-type, sub-themes were identified. The principal sub-themes were use of the Smartphone library in supporting: teaching and learning from observation; transition (from medical student to F1); trainee discourse with senior colleagues; and independent practice (supporting trainees when seniors were unavailable). The use of the device as a resource for fast access to reliable information in the workplace and the implication this had for confidence and efficient patient care was a further theme. We provide evidence for these themes. The first illustration relates to the teaching and learning from observation. In this example, the medical student used the Smartphone library to prepare for and consolidate learning:

Often within the surgical setting a student is not scrubbed in and simply observes the procedures of the day… Yet with prior knowledge of the cases it is possible to use the time between patients being in the theatre to gain further insight into the anatomy the surgeon will be encountering, the condition the patient suffers from and the surgical techniques used. Being able to find all the relevant information on my iDoc phone gave me immediate insight into each procedure and provided an improved knowledge base.

In another, complementary example, a more senior trainee (core training year 2) reported how he, as a teacher, used the iDoc device with students in the operating theatre:

Between cases, I taught two final year medical students anatomy relevant to the cases in theatre. Images from Atlas of Human Anatomy (Netter, 2003), on the Smartphone, provided visual reference.

Interestingly, this doctor reflected on how the device supported ‘situating learning’, making specific reference to Lave and Wenger [10]:

When teaching is impromptu, conventional multimedia equipment may be either unavailable or inappropriate. Describing the concept of ‘situated learning’, Lave and Wenger (1991), suggest learning is more effective when performed in an appropriate context. The portability of the Smartphone facilitated teaching anatomy in the context of its clinical application within general surgery. It provided visual stimuli to enrich several ad hoc teaching experiences in a single day.

The sub-theme of transitions is illustrated by an F1 doctor who described how the iDoc had helped him “enormously with the transitional process from medical student to F1”. In alluding to the hidden curriculum of the workplace, he explained how:

The iDoc books and information are incredibly helpful not only for answering medical questions but with the many wider jobs that an F1 doctor must be competent at. This is often pieces of information that are never actually taught or tested whilst at medical school and are relied upon to be picked up within the hospital setting.

Data related to the preparation for discourse sub-theme demonstrated how the Smartphone library was used to prime the trainees for discussion with senior colleagues. For example, an F2 trainee reported how:

The iDoc provided me with relevant information, so that I was able to articulate the urgency of the situation to the medical registrar so that this patient's management may be reviewed for escalation to ITU.

Another doctor (core training year 1, CT1) explained how she used the device to get “the answer to a question” instead of bothering “senior colleagues”. However she elaborated:

If I cannot find the answer or feel that my patient is a little more complex and needs discussion with my registrar or consultant, I can then enter into this discussion.”

How the Smartphone library complemented rather than displaced trainees’ discussion with their senior colleagues was emphasised by others:

The iDoc device does not replace the need for senior opinion in complex cases (Male, Specialist training year 1, ST1)

Seniors were not always available. They may be, for example, scrubbed in theatre, attending an emergency or remotely supervising trainees on nightshift. The case report data also provided evidence of the Smartphone library supporting trainees in circumstances when seniors were unavailable. One F2 described how the device “contributed to addressing the problem as senior advice was unavailable”. She added that:

It benefited the patient who then had correct initial treatment and investigations prior to senior review.

The final theme from the case reports that we consider here related to the value of speedy access to reliable information and how this could enhance efficiency. One F2 thought that consulting the texts (the BNF) on the Smartphone was:

Much, much quicker than flicking through the paper version… Looking things up in the paper BNF for the n-th time on ward rounds puts time pressure on the junior doctor causing stress and increasing risk of errors.

Another F2 commented:

Because I had found out information relatively quickly the patient got faster treatment and I was able to carry on with the rest of my jobs.

Analysis of the case reports showed that the Smartphone library was used for seeking information for simple (information-based) and complex (problem-based) clinical questions as well as clinical procedures (skills-based scenarios). On further analysis additional themes were identified relating to how use of the Smartphone library assisted teaching and learning from observation, transition from medical student to F1, preparing trainee doctors for discussions with their senior colleagues and as a support when seniors were unavailable. The use of the device as a resource providing ‘just-in-time’ access to reliable information which enhanced the efficiency of patient care was a further theme.

## Discussion

Given the explosion of information, reduced time for training in the UK and the increasing complexity of medicine, trainees need access to reliable information. Moore [1] argues that “research continues to show a disconnect between the information physicians need and the information they retrieve”. The iDoc project provides evidence that suggests that a Smartphone containing a searchable library of texts could be a part of the solution to these challenges. Earlier work by Prgomet et al. [7] reported that PDAs showed greatest benefits in contexts where “time is a critical factor and a rapid response crucial”. They also indicated the need for more evaluations of these technologies. Our study adds to this developing evidence base.

In summary, our data showed that a variety of information sources were consulted by trainees in the workplace on a regular basis and that ‘people based’ resources (senior colleagues, peers, other members of the medical team) were generally most popular. However, the choice of information resource varied according to the question or issue being addressed as well as the level of supervision (and notably whether senior colleagues were available). The transition phase from medical student to new doctor (F1) was seen as the most useful stage at which to provide a mobile device containing medical information. Our data showed how mobile technology was used as a ‘just-in-time’ information resource in daily clinical practice, particularly when other sources were not available, and as a resource for context-based teaching and learning in the clinical setting. This supports findings from the recent Davies et al. study [9] which suggested that, in an undergraduate clinical population, “Learning occurred in context with timely access to key facts…”. Beyond this, however, our data also showed how the Smartphone library supported the interchange between explicit and tacit knowledge. It did this by priming the junior doctors with explicit knowledge so that they were better prepared for discussions with their senior colleagues in that community of practice. The ready access to reliable, timely and accurate information supported that dialogue. Dexter and Dornan [16] write about how technology can be used to enhance the social processes of learning as well as to deliver subject matter. Such discussions can help trainees gain insight into the tacit knowledge held by the community of practice. This is the conversion process that Nonaka [11] described as internalization (explicit to tacit knowledge). The trainees learn from the dynamic interaction between explicit and tacit knowledge. Using the iDoc device to look up information can prepare trainees for a more fruitful discussion with a senior colleague and such discussion might reveal insights into their tacit knowledge.

Harper Lee’s *To Kill a Mocking Bird* novel [17] provides an excellent illustration of tacit knowledge. Set in Alabama in the 1930s when segregation was in place, one of the central themes is racism. The narrator of the story is a young girl who we see growing up. Her experience of living in the small town in the Southern States and the events that happen in the story bring her to an understanding of racism and its injustice. Yet she was not explicitly taught about racism. Rather she developed her tacit understanding of the injustice of racism through experience. What is noteworthy here is that readers of the novel also gain insight into her tacit knowledge and develop their own understanding of racism. In the iDoc project, the case reports are a form of story. The doctors authoring these ‘stories’ are making their tacit, experiential knowledge, about the application of this tool, explicit so enabling it to be shared with others. We argue that the case reports are a form of externalization, the process of converting tacit knowledge to explicit [11]. The value of *written* accounts and reflections requires further investigation which could have implications for the use of workplace based assessments and e-portfolios.

Our study has limitations. Not all in the study made use of the device and some stopped using it. There was limited up-take in the pilot phase of recruitment. Reasons related to the need to carry a device in addition to their own mobile phone, concerns about loss or theft and a dislike of the device which was seen as ‘old technology’. Davies et al. [9] identified similar concerns. Some of the reluctance however, was borne out of a lack of confidence in using Smartphones. This challenges the assumption that all young people are ‘digital natives’ and as Coulby et al. [18] suggest, we should not assume digital competence when using technology in the support of learning.

Our work may also be critiqued for limiting its focus on accessing information, overlooking other stages in the clinical decision-making process including the insight needed to identify a gap in knowledge and how to analyse and apply information [1]. However, data in the case reports detail just how the retrieved information was employed in practice. The case reports provide our richest data source. Although all in the study were invited to submit case reports, these were provided by a volunteer sample only and analysis is on-going. Further work could also be undertaken on how use of Smartphones in the workplace is perceived by staff and patients.

## Conclusion

A variety of information-sources are regularly used in the workplace. ‘People-based’ resources are important and used daily but are not always available. In times of transition, constant access to a searchable mobile library of texts is a valuable support and, recognising the social process of learning [16], offers scope to make best use of people-based discussion.

That trainees’ preference for information source varies raises questions for training. Should support be sensitive to gender, tailored to level of supervision, and more concentrated at times of transition? Do trainees need to be advised on the appropriateness of different information-sources for different types of question or problem? Can a mobile information device prepare trainees so that they are able to make better use of discussion with senior colleagues? However, we should also be atune to potential unwelcome consequences. Does technology make learners lazy (just enough and just-in-time), and does this encourage shallow learning? [19] Evidence from the iDoc evaluation refutes this suggestion, demonstrating a use of technology that complements and enriches workplace learning.

Providing access to reliable information will enhance patient care by supporting accurate prescribing and treatment planning. Case reports have shown how trainees were able more rapidly to medicate patients to reduce pain and more quickly to call for specific assessments. We do not have documented evidence on what the project participants would have done had they not accessed the Smartphone library to support their clinical decision-making. Anecdotally trainees have told us that they would have waited until seniors were free to consult or would have searched for hard copy texts, which may not have been up-to-date. These strategies would have added time which may have had serious consequences for patient care. However, a Smartphone library does not replace consultation with more senior colleagues in the community of practice. Clinical decision-making often requires dialogue and discussion. What the Smartphone library can do is augment these discussions by preparing trainees so that they can have a more informed, more confident and potentially more efficient discussion.

## Competing interests

Both the iDoc initiative and the evaluation were funded by the Wales deanery. We note that the evaluation was led from the Cardiff University School of Social Sciences. The Dr Companion software was purchased from a commercial company but they had no influence or input into the design or conduct of the evaluation.

## Authors’ contribution

The study was conceived by MS and the evaluation designed by ABu, WH and MS. Acquisition of data and data coordination was principally undertaken by WH with some assistance from ABr, SCI, AB and MS. All authors contributed to data analysis and interpretation. The manuscript was drafted by ABu and WH and other authors provided comment. All authors approved the final version.

## Pre-publication history

The pre-publication history for this paper can be accessed here:

http://www.biomedcentral.com/1472-6920/13/6/prepub

## Supplementary Material

Additional file 1 Textbooks pre-loaded on Medhand Dr Companion SD card. Click here for file

## References

[B1] Moore M the Association of Academic Health Sciences L Teaching physicians to make informed decisions in the face of uncertainty: librarians and informaticians on the health care team Acad Med 2011 86 11 1345 10.1097/ACM.0b013e3182308d7e 22030644

[B2] Davies K The information-seeking behaviour of doctors: a review of the evidence Health Info Libr J 2007 24 2 78 94 10.1111/j.1471-1842.2007.00713.x 17584211

[B3] Donaldson L Good Doctors, Safer Patients 2006 London: Department of Health

[B4] Jen MH Bottle A Majeed A Bell D Aylin P Early in-hospital mortality following trainee doctors’ first day at work PLoS One 2009 4 9 e7103 1–5 10.1371/journal.pone.0007103 19774078 PMC2743809

[B5] Teunissen PW Westerman M Opportunity or threat: the ambiguity of the consequences of transitions in medical education Med Educ 2011 45 1 51 59 10.1111/j.1365-2923.2010.03755.x 21155868

[B6] Weightman AL Williamson J The value and impact of information provided through library services for patient care: a systematic review Health Info Libr J 2005 22 1 4 25 10.1111/j.1471-1842.2005.00549.x 15810928

[B7] Prgomet M Georgiou A Westbrook JI The Impact of Mobile Handheld Technology on Hospital Physicians’ Work Practices and Patient Care: a Systematic Review. J Am Med Inform Assoc 2009 16 6 792 801 10.1197/jamia.M3215 19717793 PMC3002124

[B8] Morgan M Pugsley L Bullock A Phillips S Stacey M Evaluating trainee doctors’ educational use of a personal digital assistant: a pilot study Br J Hosp Med 2010 71 8 461 464 10.12968/hmed.2010.71.8.77670 20852489

[B9] Davies BS Rafique J Vincent TR Fairclough J Packer MH Vincent R Haq I Mobile Medical Education (MoMEd) - how mobile information resources contribute to learning for undergraduate clinical students - a mixed methods study BMC Med Educ 2012 12 1 10.1186/1472-6920-12-1 22240206 PMC3317860

[B10] Lave J Wenger E Situated learning: Legitimate peripheral participation 1991 Cambridge: Cambridge University Press

[B11] Nonaka I A dynamic theory of organizational knowledge creation Org Sci 1994 5 1 14 37 10.1287/orsc.5.1.14

[B12] Fish D De Cossart L Developing the Wise Doctor 2007 London: Royal Society of Medicine Press

[B13] Sfard A On Two Metaphors for Learning and the Dangers of Choosing Just One Educ Res 1998 27 2 4 13

[B14] Ellaway R Apples and Architraves: a Descriptive Framework for e-Learning Research Med Teach 2010 32 1 95 97 10.3109/01421590903573966 20095783

[B15] David B Yin C Chalon R Sanchez IA Contextual mobile learning for appliance mastery IADIS International Conference Mobile Learning 2007 Lisbon, Portugal: IADIS

[B16] Dexter H Dornan T Technology-enhanced learning: appraising the evidence Med Educ 2010 44 8 746 748 10.1111/j.1365-2923.2010.03758.x 20633214

[B17] Lee H To Kill a Mocking Bird 1960 New York: Grand Central Publishing

[B18] Coulby C Hennessey S Davies N Fuller R The use of mobile technology for work-based assessment: the student experience Brit J Educ Tech 2011 42 2 251 265 10.1111/j.1467-8535.2009.01022.x

[B19] Kassirer J Does access to compiled information undermone clinical cognition? Lancet 2010 376 1510 1511 10.1016/S0140-6736(10)60895-1 21036275

