# Supplementary material for: Mobile technology supporting trainee doctors’ workplace learning and patient care: an evaluation
Source: BMC Med Educ. 2013 Jan 21;13:6. doi: 10.1186/1472-6920-13-6 (PMC3552772; doi:10.1186/1472-6920-13-6)
Supplement: Additional file 1 — Textbooks pre-loaded on Medhand Dr Companion SD card. [file 1472-6920-13-6-S1.docx]

**Additional information**

Textbooks pre-loaded on Medhand Dr Companion SD card

1. BNF
2. Chemical Laboratory references
3. Classification of Surgical Operations and Procedures (OPCS 4)

4. Clinical Evidence

1. Cochrane Abstracts
2. Harrison’s Manual of Medicine
3. International Classification of Diseases 10 (ICD 10)
4. Netter’s Atlas of Human Anatomy
5. NICE Guidance Compilation
6. Oxford Handbook for the Foundation Programme
7. Oxford Handbook of Clinical Laboratory Investigations
8. Oxford Handbook of Clinical Medicine
9. Oxford Handbook of Clinical Specialties
10. Oxford Handbook of Clinical Surgery
11. Register of Patient Organisations in the UK
12. Stedman's Medical Dictionary
13. Stockley's Drug Interactions
